# Streamlining feature elaboration and statistics analysis in metabolomics: the GetFeatistics R-package

**DOI:** 10.1515/jib-2025-0047

**Published:** 2025-12-24

**Authors:** Gianfranco Frigerio

**Affiliations:** Center for Omics Sciences (COSR), IRCCS San Raffaele Scientific Institute, Milan, Italy; Luxembourg Centre for Systems Biomedicine (LCSB), University of Luxembourg, Belvaux, Luxembourg; Department of Clinical Sciences and Community Health, University of Milan, and Fondazione IRCCS Ca’ Granda Ospedale Maggiore Policlinico, Milan, Italy

**Keywords:** metabolomics workflow, non-targeted metabolomics, computational metabolomics, open-source software, data preprocessing

## Abstract

Metabolomics studies require complex data processing pipelines to ensure data quality and extract meaningful biological insights. GetFeatistics is an R-package developed to streamline the elaboration and statistical analysis of metabolomics data. For targeted analyses, the package enables calibration curve-based quantification with different data weighting options. For untargeted studies, it includes dedicated functions to import feature tables from tools like patRoon and MS-DIAL, assign annotation confidence levels, and filter features based on pooled quality control (QC) criteria, including options for group-specific pooled QCs. The package also provides functions for univariate and multivariate statistical analyses, notably streamlined regression modelling with fixed effects, mixed-effects models for longitudinal data, and Tobit regression for censoring values exceeding the limits of detection. Output tables are concise and informative, facilitating interpretation and reporting, while output visualisations are fully customisable via the ggplot grammar. Additional functionalities include automated retrieval of chemical properties from PubChem, ontology classification via ClassyFire, and pathway enrichment analysis using the FELLA package. GetFeatistics is publicly available on GitHub, with comprehensive documentation and a step-by-step vignette. By integrating key steps of the metabolomics workflow, the package aims to facilitate both exploratory studies and large-scale epidemiological applications in metabolomics research.

## 1 Introduction

Metabolomics is the youngest of the four main “omics” sciences – following genomics, transcriptomics, and proteomics – and as such, still requires methodological development and improvement. Metabolomic approaches can be broadly categorised into targeted and untargeted strategies. Targeted metabolomics aims to accurately quantify a predefined set of metabolites, whereas untargeted metabolomics seeks to capture the widest possible spectrum of metabolites to explore biological differences without prior assumptions.

In untargeted workflows, the inclusion of pooled quality control (QC) samples during sample preparation is a key step to monitor data quality and analytical reproducibility throughout the acquisition process [1]. Community-driven initiatives such as the Metabolomics Quality Assurance and Quality Control Consortium (mQACC) are actively working to promote the implementation of QC practices in metabolomics studies [2]. In addition, the use of group-specific QC samples has been proposed to prevent the dilution of low-abundance metabolites that may fall below the instrument’s detection limit when using a single pooled QC for all groups [3].

Following instrumental analysis with liquid chromatography coupled to high resolution mass spectrometry (LC-MS/MS), data elaboration is usually the most time-consuming and demanding step of the metabolomics workflow. Open-source tools such as XCMS [4] are used to extract metabolic features from raw data files, where a feature is defined as peak with specific mass-to-charge ratio (*m*/*z*) and retention time (*rt*); additional tools like CAMERA [5] assist with the grouping of adducts and isotopes; while annotation software such as MetFrag [6] and databases like PubChemLite [7] support metabolite annotation: all of these have being integrated into the patRoon R package [8], 9]. An alternative widely adopted solution is MS-DIAL, a user-friendly software that spans the same pipeline from feature extraction to annotation, the latter made possible thanks to the extensive publicly available MSP libraries [10], 11].

Once features have been extracted and potentially annotated, filtering based on QC quality cut-off criteria is typically performed; data intensities are pre-processed to deal with missing values and data normality; and then statistical analyses are applied to highlight biologically relevant associations. Since the metabolome is influenced by several factors, it is essential to properly account for potential confounding variables, particularly in observational epidemiological studies, and this can be achieved by applying regression models with multiple covariates. For longitudinal designs, mixed-effects models that incorporate both fixed and random effects are a suitable approach [12], 13]. In the context of targeted analyses, Tobit regression models are a suitable approach to handle censored data, i.e.: metabolite concentrations that fall below or above quantification limits [14], 15].

Presented here is the R-package “GetFeatistics” (feature and statistics). The tool is designed to streamline the data elaboration of targeted and untargeted metabolomics studies, by offering an R-based pipeline for data processing, particularly for pooled QCs filtration, and statistical analyses, notably mixed effect and Tobit regression models.

## 2 Implementation and workflow

**Figure 1:**
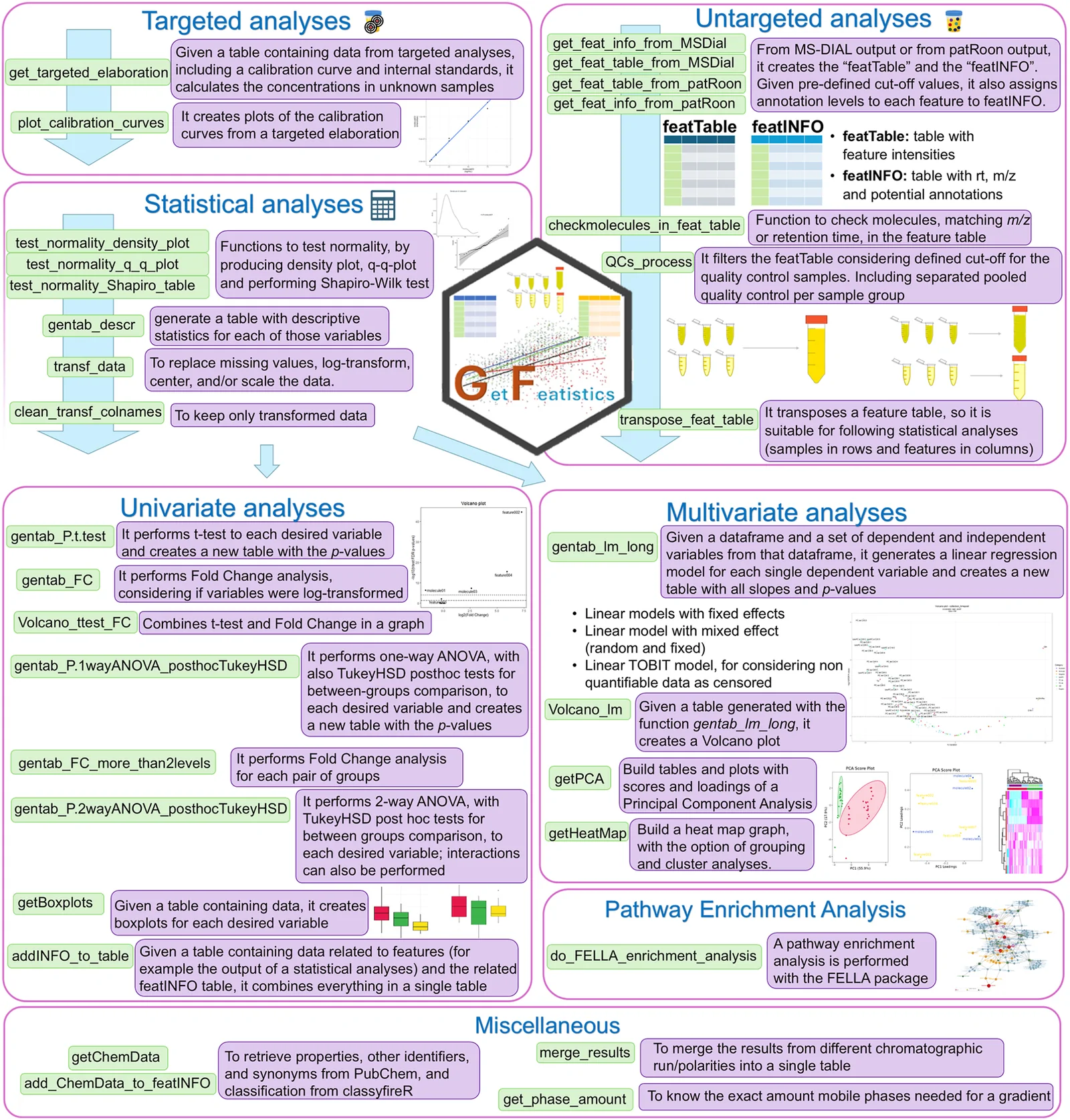
Typical workflow for data elaboration that can be implemented with the GetFeatistics R-package. Each green rectangle represents a function, and each purple rectangle contains a brief description of it. The logo of the package is reported in the centre of the picture. Example graphs were generated using mock data and from a previously published study under the author’s retained rights [13].

The GetFeatistics package has been developed with the R-language and is publicly available on GitHub. Most of its functions rely on the tidyverse ecosystem [16], and all the visualisations are created as “ggplot” objects, thus allowing further customisation with the ggplot grammar. The website of the package, built using the pkgdown framework [17], provides extensive documentation for each function, and the vignette shows the application of the workflow on sample data. Figure 1 illustrates the overall data processing workflow, highlighting the functions involved in each step.

### 2.1 Targeted analyses

The package provides straightforward calculation of absolute concentrations from signal intensities via the function *get_targeted_elaboration*. The function’s arguments include: (I) a table of intensities for samples, quality controls, and calibration standards used to build the curve; (II) a table reporting the nominal concentrations for quality controls and calibration levels; and (III) optionally, a table mapping each analyte to its internal standard and specifying the desired weighting scheme. Because calibration data often have higher variance at higher concentrations, applying weighted least squares (e.g., 1/*Y*, 1/*X* or 1/*X*^2^), reduces bias and improves accuracy at low concentrations [18], 19]. The function returns calculated absolute concentrations and accuracy (%) for standards and quality controls. These results can be passed to *plot_calibration_curves* to visualise the fitted calibration curves.

### 2.2 Untargeted analyses

Following feature extraction, grouping, and annotation using external tools such as patRoon or MS-DIAL, the workflow of GetFeatistics is then based to be conducted on two core data frames: “featTable”, where rows represent features and columns represent samples, containing intensity values; and “featINFO”, where rows again represent features, while columns contain the feature metadata such as *m*/*z*, retention time, chemical annotations, and other descriptors of the feature or of the annotated molecule. Dedicated functions are available to import and structure these data frames from patRoon (*get_feat_info_from_patRoon*, *get_feat_table_from_patRoon*) and MS-DIAL outputs (*get_feat_info_from_MSDial*, *get_feat_table_from_MSDial*). Confidence levels of annotated compounds can be automatically assigned according to the annotation grading proposed by Schymanski et al. [20], particularly implementing the MoNAScore cut-offs, for patRoon, and the dot product and fragment presence cut-offs, for MS-DIAL, as reported by Talavera Andújar et al. [21]. Known molecules can be searched in the feature list based on their exact mass; moreover, if the retention time is also available, i.e.: when analytical standards have been analysed under the same instrumental conditions, matching features can be identified and a confidence level 1 is then assigned (*checkmolecules_in_feat_table*).

In order to improve the quality of the dataset and retain only biologically meaningful features, the package includes a function to filter features based on QC criteria (*QCs_proces*) by filtering out features with: (I) poor reproducibility in pooled QCs (based on relative standard deviation of QC intensities); (II) insufficient presence across QC injections (based on detection frequency in QCs); (III) high blank contribution (based on blank/QC mean intensity ratio); (IIII) unreasonable response in diluted QC samples (based on a range of acceptability of QC/diluted QC mean intensity ratio). When group-specific QC pools are available, the filtering retains features meeting quality thresholds in at least one group [3].

### 2.3 Statistical analyses

A summary table with descriptive statistics can be generated (*gentab_descr*) considering the sample groups. Normality is assessed via density plots (*test_normality_density_plot*), Q–Q plots (*test_normality_q_q_plot*), and the Shapiro–Wilk test (*test_normality_Shapiro_table*). Data can then be processed via missing value imputation, log-transformation, centering, and scaling (*transf_data*). A suite of functions supports univariate statistical testing, including *t*-tests (*gentab_P.t.test*), fold change analysis (*gentab_FC*, *gentab_FC_more_than2levels*), one- and two-way ANOVA, with or without interaction terms (*gentab_P.1wayANOVA_posthocTukeyHSD*, *gentab_P.2wayANOVA_posthocTukeyHSD*). Considering the application of such statistical tests on several metabolites, *p*-values can be corrected using false discovery rate (FDR) procedures [22]. Results can be visualized through volcano plots (*Volcano_ttest_FC*) and box plots (*getBoxplots*). The outcome tables from statistical analyses can be enriched with corresponding feature metadata (*addINFO_to_table*).

For multivariate analysis, a single function (*gentab_lm_long*) allows the construction of regression models across the entire metabolite dataset, incorporating one or more covariates, with the option of building linear models with fixed effects, mixed-effects models with the “lmerTest” package [23], 24], or Tobit regression for censored data (specifically suited in this workflow for values below or above detection limits) via the “AER” package [25]. The output of the function is a table with complete effect estimates, with related confidence intervals and *p*-values (with also FDR correction), for each metabolite – covariate pair. Visual summaries of results through a Volcano plot can be created (*Volcano_lm*). Principal component analyses and heatmap with hierarchical clustering can be also performed with extensive customisation options (*getPCA*, *getHeatMap*).

### 2.4 Miscellaneous

The package also contains functions (*getChemData*, *add_ChemData_to_featINFO*) to automatically retrieve chemical properties, synonyms, and identifiers from PubChem using the Power User Gateway (PUG) [26], and chemical ontology classification via the ClassyFire taxonomy [27]. Additionally, a function (*do_FELLA_enrichment_analysis*) is provided for pathway enrichment analysis using the FELLA package [28].

Finally, the package includes a functionality (*merge_results*) to merge results from multiple chromatographic runs and/or ionization modes, prioritising overlapping compounds according to user-defined criteria (e.g., lower mass error, retention time shift, or *p*-value).

## 3 Conclusions

In conclusion, the GetFeatistics R-package streamlines the processing and statistical analysis of both targeted and untargeted metabolomics data. It integrates a wide range of functionalities, and the novelty rely particularly in the filtration of features considering pooled QC criteria, including also the use separated pooled QC per sample group, and the efficient construction of multiple regression models with mixed-effect and Tobit models. The package is publicly available, fully documented, actively maintained, and open to contributions from the community.
